# Purification and interactions of the MucA’ and MucB proteins constituting the DNA polymerase RI

**DOI:** 10.1186/s41021-019-0125-8

**Published:** 2019-05-02

**Authors:** Petr Grúz, Kei-ichi Sugiyama, Masamitsu Honma, Takehiko Nohmi

**Affiliations:** Division of Genetics and Mutagenesis, National Institute of Health Sciences, 3-25-26 Tonomachi, Kawasaki-ku, Kawasaki-shi, Kanagawa 210-9501 Japan

**Keywords:** DNA polymerase, mucAB, Surface Plasmon resonance, Protein refolding, β-Subunit

## Abstract

**Background:**

The MucA’ and MucB proteins comprise the core of DNA polymerase RI which is a strong mutator utilized in mutagenicity assays such as the standard Ames test. A close relative DNA polymerase V, composed of the homologous UmuD’ and UmuC proteins, is considered to be an ortholog of the mammalian DNA polymerase η. The catalytic subunits of these polymerases belong to the Y-family which specializes in the translesion DNA synthesis across various DNA adducts to rescue stalled chromosomal replication at the expense of mutations. Based on genetic evidence, DNA polymerase RI possesses the greatest ability to induce various types of mutations among all so far characterized members of the Y-superfamily. The exceptionally high mutagenic potential of MucA’B has been taken advantage of in numerous bacterial mutagenicity assays incorporating the conjugative plasmid pKM101 carrying the *mucAB* operon such as the Ames Test.

**Results:**

We established new procedures for the purification of MucB protein as well as its accessory protein MucA’ using the refolding techniques. The purified MucA’ protein behaved as a molecular dimer which was fully stable in solution. The soluble monomeric form of MucB protein was obtained after refolding on a gel-filtration column and remained stable in a nondenaturing buffer containing protein aggregation inhibitors. Using the surface plasmon resonance technique, we demonstrated that the purified MucA’ and MucB proteins interacted and that MucB protein preferentially bound to single-stranded DNA. In addition, we revealed that MucB protein interacted with the β-subunit of DNA polymerase III holoenzyme of *E. coli*.

**Conclusion:**

The MucA’ and MucB proteins can be isolated from inclusion bodies and solubilized in vitro. The refolded MucB protein interacts with its MucA’ partner as well as with DNA what suggests it retains biological activity. The interaction of MucB with the processivity subunit of DNA polymerase III may imply the role of the subunit as an accessory protein to MucB during the translesion DNA synthesis.

**Electronic supplementary material:**

The online version of this article (10.1186/s41021-019-0125-8) contains supplementary material, which is available to authorized users.

## Introduction

In *Escherichia coli,* mutagenesis by ultraviolet light and most chemicals requires the expression of the *umuDC* operon [1, 2]. The *umuDC* operon is located at about 26 min on the *E. coli* chromosome and encodes the 15.1- and 47.7-kDa proteins UmuD and UmuC, respectively [3, 4]. Expression of the *umuDC* operon and other homologous operons, such as *mucAB, impAB, samAB* and *rumAB*, is regulated as a part of SOS response of *E. coli*, in which an activated form of RecA mediates the cleavage of the repressor protein LexA [5–9]. When SOS response is turned on, the intracellular levels of UmuDC proteins raise to as high as 2400 and 200 molecules of UmuD and UmuC proteins, respectively, from 180 molecules of UmuD and undetectable amounts of UmuC under uninduced conditions [10]. The activated form of RecA also mediates the cleavage of UmuD [11–13]. The resulting carboxy-terminal fragment of UmuD, i.e., UmuD’, is necessary and sufficient for the role of UmuD in UV mutagenesis [12]. UmuD’ forms a homodimer that makes a complex with UmuC [14]. The appearance of the UmuD’C protein complex switches DNA repair from homologous recombination to SOS mutagenesis [15]. Besides mediating the cleavage of LexA and UmuD, RecA plays another essential role in UV mutagenesis in *E. coli* [12, 16]*.* The most plausible hypothesis for the third role of RecA in UV mutagenesis is that RecA interacts with UmuD’ or UmuC, thereby targeting the UmuD’C complex to lesions in DNA [17–19]. It has been shown that the UmuC protein has an intrinsic DNA polymerase activity dependent on the accessory proteins UmuD’, RecA*, Ssb and β, γ complex [20, 21]. This DNA polymerase has been named Pol V next to another homologous previously characterized DNA polymerase Pol IV encoded by the *din*B gene and involved in untargeted mutagenesis [20, 22]. In accordance with the in vivo data, Pol V is a highly *error-prone* DNA polymerase which efficiently bypasses abasic sites and other DNA lesions [20] and has been considered to be an ortholog of the mammalian DNA polymerase η [23].

Despite the recent progress in elucidating the structures of UmuD’ monomer [24] and UmuD’ dimer [25] and getting deeper insights into the interactions between UmuD and RecA* [26], the structure and biochemistry of the UmuC protein and its close homologues still remains largely unknown. The purification of the UmuC protein has been difficult due to its high instability in solution. It was first purified from a denatured form and renatured in the presence of chaperone proteins [14, 27]. Using the glycerol gradient sedimentation analysis, it has been shown that UmuC protein exists as a monomer in solution and forms a complex with UmuD’ corresponding to two UmuD’ and one UmuC associated molecules [14]. Interaction between UmuD’ and UmuC proteins has been demonstrated by the immunoprecipitation techniques [27] and the yeast two-hybrid system [28]. Using RecA* affinity column chromatography, it has been shown that UmuC protein interacts with RecA* [19]. The MucB protein, a close homologue of UmuC, purified from inclusion bodies by Livneh et al. [29] was shown to interact with single strand DNA binding protein (SSB). Finally, the UmuC protein has been purified in a soluble form either in a complex with UmuD’ [30] or as a fusion to maltose binding protein (MBP) [31] and used to demonstrate its intrinsic DNA polymerase activity.

In order to help understanding the molecular basis of translesion DNA synthesis by the Y-family DNA polymerases [32, 33], we chose to study the MucA’B proteins. The gene products of *mucAB* possess the highest ability to promote various types of mutagenesis in vivo among all so far characterized *umuDC*-like operons [34]. The experiments with single-stranded vector carrying specifically located abasic sites confirmed that the MucA’B proteins have inherently the greatest capacity to promote translesion DNA synthesis [35]. They also appear more stable than the UmuDC proteins because of their independence on the molecular chaperones in vivo [36]*.* Because of the remarkably higher mutagenic potential of the *mucAB* operon and its involvement in the widest range of different types of mutagenesis, we anticipate that the active form of MucA’B, homologous to the DNA polymerase V, will be the best subject for the biochemical study of DNA synthesis through various chemically induced DNA lesions. In this paper, we present new methods for the separate purification of MucA’ and MucB proteins by in vitro refolding from inclusion bodies. The purified MucB protein interacted with MucA’, RecA and single-stranded DNA (ssDNA). In addition, we found that the purified MucB protein interacts with β subunit of DNA polymerase III holoenzyme of *E. coli*. The implications of these interactions for the translesion DNA synthesis are discussed.

## Materials and methods

### Materials

The sources of chemicals used in this study were as follows: Lysozyme from Merck, NJ; Tween 20 used for MucB refolding was purchased as a specially purified 10% aqueous solution from Pierce, IL; other chemicals were of analytical grade and were purchased from Wako Pure Chemicals, Osaka, Japan. The reagents such as Surfactant P20 and sensor chips used for surface plasmon resonance (SPR) experiments performed on the BIAcore™ 2000 instrument were purchased from Biacore AB, Uppsala, Sweden. The β-subunit of DNA Polymerse III holoenzyme [37] was obtained from Toyobo, Tokyo, Japan. The purity of β-subunit was greater than 95% judged by sodium-dodecyl-sulfate polyacrylamide gel electrophoresis (SDS-PAGE) and it existed in the form of a molecular dimer as confirmed by gel filtration on Superdex 200 PC 3.2/30 column (Pharmacia, Sweden). Bovine serum albumin (BSA), RecA and single-stranded DNA binding protein (SSB) proteins were purchased from Pharmacia, Sweden, and their purity was greater than 95% as judged by SDS-PAGE analysis. The ability of RecA protein to bind DNA in the presence of Mg^++^ cations was confirmed using the BIAcore 2000 instrument and streptavidine-coated SA chip with captured oligonucleotide. The SDS-PAGE molecular weight standards were purchased from Bio-Rad Laboratories, CA, and the gel filtration molecular weight markers were from Sigma, MO. The prepacked columns Superdex 75 XK 16/60 and Superdex 200 HR 10/30 were purchased from Pharmacia, Sweden. MucA’ polyclonal antiserum was raised against MucA’ protein purified from inclusion bodies by preparative SDS-PAGE in rabbits by TaKaRa Shuzo, Kyoto, Japan.

### Bacterial strains and plasmids

The plasmids pYG8506 and pYG8512 used for the overproduction of MucA’ and MucB proteins, respectively, were constructed as described previously [38]. The strains used for overexpression were constructed by introducing these plasmids into *E. coli* strains BL21(DE3) and BL21(DE3)/pLysS [39] by standard transformation techniques.

### Purification of MucA’ protein

Overnight culture of strain BL21(DE3)/pYG8506 grown in 50 ml of M9 minimal medium supplemented with 70 μg/ml ampicillin was washed once in 40 ml of 2xYT medium and used to inoculate 500 ml of 2xYT medium supplemented with 50 μg/ml ampicillin and prewarmed to 37 °C. The culture was incubated at 37 °C for 1.5 h with shaking and then the overexpression of MucA’ protein was induced by adding IPTG to a final concentration of 1 mM. After additional incubation for 1 h, rifampicin was added to a final concentration of 200 μg/ml and incubation continued for another 5 h. Cell harvest and purification of the inclusion bodies were carried out as described by Lin and Cheng [40] with the exception that lysozyme (at final conc. 100 μg/ml) was added together with the protease inhibitors and the purified inclusion body pellet containing about 70–80 mg of MucA’ was dissolved in 10 ml of buffer D (50 mM Tris buffer pH 8.0, 5 mM EDTA, 6 M guanidium hydrochloride, 10 mM DTT). The undissolved contaminants were then removed by centrifugation and passing the supernatant through 0.45 μm filter. The total volume was adjusted to 15 ml with buffer D and the solution was stored at − 20 °C until proceeding to the refolding step.

The denatured MucA’ protein (10 ml) was refolded by stepwise addition (in three 1-h intervals) into 900 ml of stirring refolding buffer R (50 mM Tris buffer pH 8.0, 10% glycerol *v*/v, 0.1 mM EDTA, 1 mM DTT) at 4 °C. After the last addition, the solution was incubated for at least 1 h at 4 °C. The re-aggregated portion of MucA’ was removed by centrifugation at 18,500 x g (it can be repeatedly used for refolding after denaturation in buffer D) and the volume of supernatant was measured. Soluble MucA’ protein was precipitated by slow addition of 243 mg of (NH_4_)_2_SO_4_ per ml of supernatant [18]. The precipitated protein was collected by centrifugation and redissolved in 1/10 of the original volume of refolding buffer R. Undissolved components were removed by centrifugation and the supernatant containing soluble MucA’ was concentrated 10–20 x in Centriprep™ 10 concentrator (Amicon Inc., MA) yielding about 5 mg of protein per 2 ml. The MucA’ protein (1 ml of 2.5 mg/ml) was then applied to the gel filtration column Superdex 75 XK 16/60 connected to the FPLC system (Pharmacia, Sweden) equilibrated with 20 mM HEPES pH 7.4, 10% glycerol *w*/w, 150 mM NaCl, 0.1 mM EDTA and 1 mM DTT at a flow rate 0.8 ml/min. The peak of MucA’ dimer eluting between 66 and 70.6 ml was collected, concentrated by Centricon™ 10 concentrator, flash frozen in liquid nitrogen and stored frozen at − 70 °C.

### Purification of MucB protein

MucB protein was overexpressed as described for the overexpression of MucA’ except that strain BL21(DE3)/pLysS+pYG8512 was used, and the M9 medium was supplemented with 70 μg/ml ampicillin and 30 μg/ml of chloramphenicol. Cells were harvested, washed once in ice-cold STE, resuspended in a total volume of 10 ml of buffer P (PBS containing 5 mM EDTA) and frozen at − 70 °C. After the cells were melted, they were processed as described for the purification of inclusion bodies containing MucA’ except that lysozyme was not added because of the presence of intracellular T7 lysozyme encoded by the plasmid pLysS. Finally, the pellet containing about 50–70 mg of relatively pure aggregated MucB protein was dissolved in 10 ml of buffer C1 (20 mM Tris pH 7.4, 200 mM NaCl, 2 mM EDTA, 6 M guanidinum hydrochloride, 10 mM DTT). The undissolved components were removed by centrifugation and the supernatant was passed through a 0.45 μm filter. The total volume was then adjusted to 15 ml with buffer C1 and the solution was stored at − 20 °C before further use.

To refold MucB, 0.1 ml of the denatured stock containing 2.64 mg/ml protein was injected on the Superdex 200 HR 10/30 column equilibrated with NAT refolding buffer (20 mM HEPES, 100 mM monopotassium glutamate, 500 mM L-arginine, 10% glycerol *w*/*v*, 2 mM DTT, 0.1 mM EDTA, 0.5% Tween 20, pH 7.3 adjusted with acetic acid) and connected to the FPLC system. Fractions corresponding to the major peak eluting between 12.4 and 13.6 ml were collected and used for further analysis or concentrated 3–10 times by the Centricon™ 10 concentrator (Amicon Inc., MA) at 4 °C, flash frozen in liquid nitrogen and stored at − 70 °C.

### Protein measurements

Protein concentrations were determined using the Protein Assay Dye Reagent from Bio-Rad Laboratories, CA, according to the manufacturer’s instructions. In all measurements, BSA was used as a calibration standard. The concentration of the refolded MucB protein was determined by a modified Bradford assay [41] since refolding buffer NAT was incompatible with the standard assay.

### SPR analysis

Immobilization of proteins was performed on the carboxymethyldextran matrix-coated sensor chip CM5 by carbodiimide covalent linkage following the manufacturers’s instructions (Amine Coupling Kit, Biacore AB). Prior to immobilization, the proteins were dialyzed at concentration 1 mg/ml against 20 mM HEPES pH 7.4, 10% glycerol (*v*/*w*), 0.1 mM EDTA, 1 mM DTT and then diluted more then 10 fold with 10 mM sodium acetate buffer at the following pH values: BSA, pH 4.6; RecA, pH 4.4; DNA polymerase III β subunit, pH 4.2; and MucA’, pH 5.0. The immobilization was carried out by injecting proteins over the activated chip surface using following contact times and concentrations: BSA, 5 min and 10 μg/ml; RecA, 6 min and 10 μg/ml; β, 6 min and 100 μg/ml; and MucA’, 2 min and 50 μg/ml.

For the analysis of MucB interaction with ssDNA, a synthetic 73-mer oligonucleotide linked at its 5′-end to biotin was used. The sequence was 5′- GCGGCGGTTGAGTAGCTCTTCTTCCAGCACGTTTTCGCCGATAATACCGGGATCGACCACGCCATCCATTACC -3′. The oligonucleotide was immobilized by passing it dissolved in HBS buffer containing 0.5 M NaCl at the oligonucleotide concentration of 0.3 μM over the streptavidin coated SA chip surface previously conditioned with 6 pulses of 50 mM NaOH with a contact time of 10 min. This resulted in a capture of approximately 1,500 RU of the 73-mer via the biotin-streptavidin coupling.

The SPR analysis of MucB protein binding was performed at a flow rate of 30 μl/min using the standard HBS or the ssDNA binding buffer (20 mM HEPES pH 8.0, 50 mM monopotassium glutamate, 10 mM magnesium acetate, 5% glycerol *w*/*v*, 0.1 mM EDTA, 2 mM DTT, 0.05% *v*/v Surfactant P20) as a running buffer for protein-to-protein or protein-to-DNA interaction analysis, respectively. For the analysis, the refolded MucB protein was diluted usually to a concentration of 0.1 μM in the running buffer containing 1% v/v Surfactant P20 if not stated otherwise in the text. When MucB was premixed with MucA’ or β, it was diluted in the running buffer containing 1% v/v Surfactant P20 plus the MucA’ or β protein at the indicated concentrations. The diluted MucB was immediately injected over the chip surface for analysis. The association rate (*k*_a_) and dissociation rate (*k*_d_) constants were determined through nonlinear curve fitting using the Pharmacia Biosensor kinetics software BIAevaluation 2.1 (Biacore AB, Pharmacia).

## Results

### Purification of MucA’ protein

**Fig. 1:**
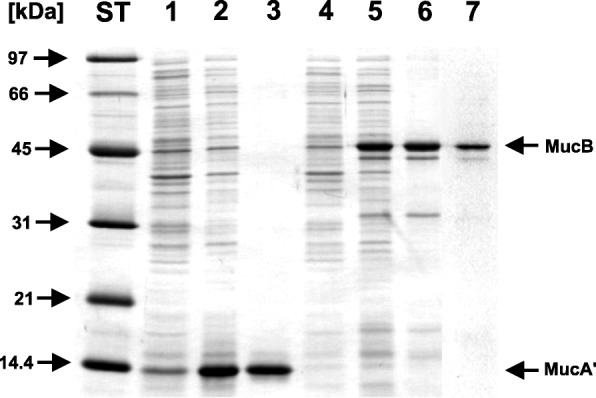
Overexpression and purification of MucA’ and MucB proteins. Proteins from samples corresponding to 20 μl of bacterial cultures or equivalent amount of protein from the purification steps were separated on a 12% SDS-polyacrylamide gel and stained with Coomassie Brilliant Blue R 250. *Lane ST*, molecular weight markers; *lane 1*, culture of the strain BL21(DE3)/pYG8506 (MucA’) before induction; *lane 2*, culture of the strain BL21(DE3)/pYG8506 (MucA’) after induction; *lane 3*, the purified MucA’ protein after gel filtration on a Superdex XK 16/60 column; *lane 4*, culture of the strain BL21(DE3)/pLysS+pYG8512 (MucB) before induction; *lane 5*, culture of the strain BL21(DE3)/pLysS+pYG8512 (MucB) after induction; *lane 6*, denatured MucB protein prior to refolding by gel filtration; *line 7*, the purified MucB protein (peak fraction after refolding by gel filtration)

We have used the previously engineered expression system based on the pET-16b vector to overproduce MucA’ protein [38]. This allowed us to reach constantly more than 30% of total cellular protein overexpression. With such a high expression most of the MucA’ protein accumulated in inclusion bodies what proved useful during early stages of purification because we could easily isolate concentrated and relatively pure protein resistant to proteolysis and readily detectable by SDS-PAGE. Due to lack of an obvious enzyme assay, we followed MucA’ only by SDS-PAGE throughout the purification (Fig. 1). The plasmid pLysS expressing T7 lysozyme, which is the natural inhibitor of T7 RNA polymerase, is used in strains expressing toxic proteins to keep the T7 RNA polymerase activity down prior to induction [39]. We did not use this plasmid in the strain overexpressing MucA’ protein because MucA’ was not toxic to the host strain and we experienced unwanted preliminary lysis of the spheroplasts containing MucA’ inclusion bodies during the purification when the plasmid was present.

**Fig. 2:**
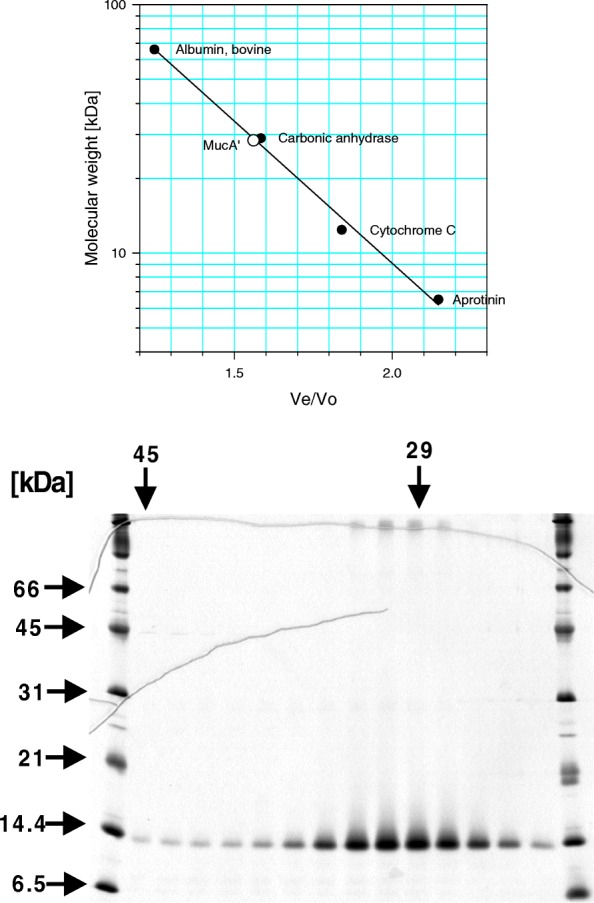
Size analysis of refolded MucA’ protein. The purified MucA’ protein was gel-filtered on a Superdex 75 XK 16/60 column connected to the FPLC system. Upper: The apparent molecular weight of MucA’ protein was estimated based on the calibration curve obtained from separate runs of protein standards from the MW-GF-70 kit according to the manufacturers’ instructions (Sigma, MO). The molecular masses of standards are as follows: Bovine serum albumin, 66 kDa; carbonic anhydrase, 29 kDa; cytochrome C, 12.4 kDa; aprotinin, 6.5 kDa. The calculated molecular weight of MucA’ monomer is 13.6 kDa. Lower: Analysis of fractions eluted from the column on a 15% SDS-polyacrylamide gel stained with Coomassie Brilliant Blue R 250. The migration of molecular weight standards in the SDS-polyacrylamide gel is shown on the left. The mobility of molecular weight markers of the indicated molecular weights in the column, as determined in a separate run, is shown on the top of the gel

The MucA’ protein in inclusion bodies appeared to be relatively stable, so that the use of the protease inhibitors during the purification was rather optional. In order to increase the yield of properly refolded MucA’ protein, we conducted its renaturation by stepwise dilution into the refolding buffer R in 1 h intervals [42]. About a half of the originally added MucA’ protein remained soluble. After clearing the solution by centrifugation, MucA’ protein was precipitated by ammonium sulfate. The precipitate was subsequently redissolved in a smaller volume of buffer R and further concentrated to about 2 mg/ml by ultrafiltration. The concentrated protein was then applied to a gel filtration column for purification and for the exchange of buffer. More than 90% of the applied protein recovered as a dimer judged by gel filtration chromatography and SDS-PAGE analysis (Fig. 2). Fractions corresponding to the MucA’ dimer peak were pooled, concentrated to at least 1 mg/ml by ultrafiltration and flash frozen in liquid nitrogen. Using this method, we were reproducibly obtaining high quality MucA’ protein with an estimated yield of at least 1.25 mg of purified MucA’ dimer per 500 ml of the induced culture containing about 130 mg of total protein.

### Purification of MucB protein

The purification of MucB protein was similar to the purification of MucA’ protein up to the refolding step with the following differences. The strain for overproduction contained the pLysS plasmid to alleviate a growth inhibition effect caused by leaking expression of the MucB protein. Because of the presence of the T7 lysozyme encoded by the plasmid pLysS, the cells could be simply lysed by freezing-and-thawing and the removal of outer cell walls and periplasm by osmotic shock was not carried out. The overexpression level of MucB protein was similar to that of MucA’, so that MucB inclusion body containing relatively pure protein could be easily isolated (Fig. 1).

Our original attempts to refold the denatured MucB protein by conventional methods such as slow dialysis against nondenaturing buffer or rapid dilution into a refolding buffer as in the case of MucA’ protein resulted in the formation of only highly insoluble aggregates. Since neither decreasing the expression rate, stimulating the culture into overproduction of chaperones (by a heat shock or by overexpression of DnaK or GroE chaperones from a separate plasmid) nor the expression of engineered N-terminal HisTag- or GST-MucB fusions led to the production of soluble MucB protein, we focused on the development of a suitable refolding buffer which would inhibit the aggregation of MucB protein upon its dilution from the denaturant. Among the wide range of tested buffers, different pH values and buffer additives, we found that low pH (citrate-phosphate buffer pH 3), L-arginine hydrochloride and nonionic detergents, such as laurylmaltoside, Tween 20 and Triton X-100, efficiently inhibited the aggregation of MucB protein. We are not counting the ionic detergents such as sarkosyl or SDS, which also prevented aggregation of MucB, here because of their denaturing nature. All the mentioned compounds, however, only slowed down the aggregation process because the MucB preparation remaining soluble for 1 h on ice still completely aggregated after a subsequent overnight storage at 4 °C. Since the protein aggregation inhibitors like arginine and the non-ionic detergents like Tween 20 act by different mechanisms to stabilize the protein in solution (see the discussion section), we postulated that they might have additive effects when used together. This assumption turned out to be indeed true because the MucB protein diluted into a buffer containing both 1% Tween 20 and 0.5 M L-arginine hydrochloride remained soluble at 4 °C over several days.

**Fig. 3:**
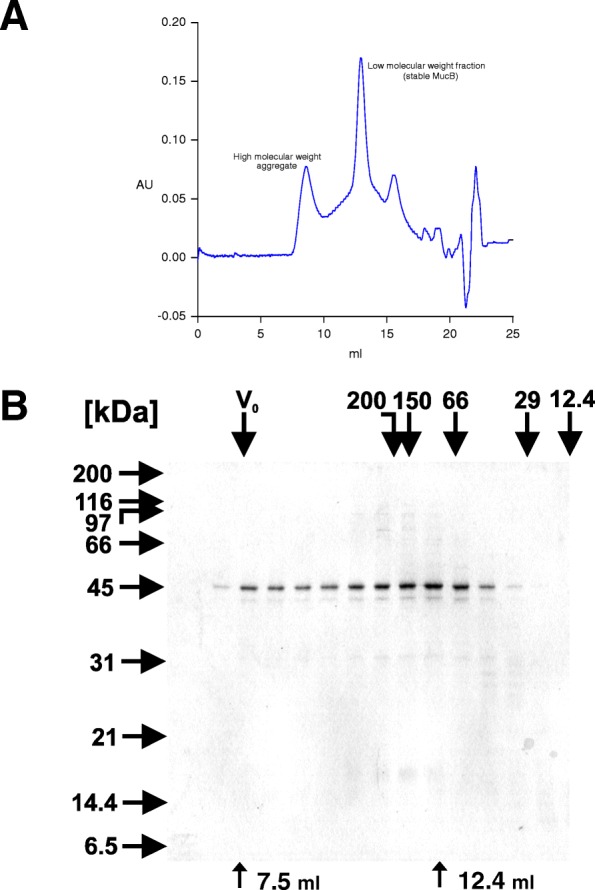
Refolding of MucB protein on a gel filtration column. The denatured MucB protein was injected on a Superdex 200 HR 10/30 column equilibrated with the NAT refolding buffer and connected to the FPLC system. **a** elution profile from the column monitored by absorbance at 280 nm. **b** analysis of fractions eluted from the column on a 12% SDS-polyacrylamide gel stained with Coomassie Brilliant Blue R 250. The migration of molecular weight standards in the SDS-polyacrylamide gel is shown on the left. The mobility of molecular weight markers of the indicated molecular weights in the column, as determined in a separate run, is shown on the top of the gel. V_0_ is void volume. The molecular weight markers are as follows: β-amylase, 200 kDa; alcohol dehydrogenase, 150 kDa; bovine serum albumin, 66 kDa; carbonic anhydrase, 29 kDa; cytochrome C, 12.4 kDa. The fractions corresponding to the column void volume and the elution volume of the collected MucB peak (eluting between 12.4 and 13.6 mls) are labeled with arrows at the bottom of the gel

Refolding of proteins during gel filtration is an effective method which allows simultaneous refolding and separation of different complexes formed during the refolding process [43, 44]. In order to improve the refolding method and get more information about the renatured MucB protein, we conducted its refolding on a gel filtration column. When the denatured MucB protein was applied to the column, we observed formation of two major peaks (Fig. 3). The first peak eluting at the void volume represented a high molecular weight (HMW) aggregate. Fractions corresponding to this HMW peak aggregated shortly after eluting from the column forming a flocculent precipitate. Fractions corresponding to the second peak representing a low molecular weight (LMW) form contained soluble apparently monomeric MucB protein at the concentration of about 25 μg/ml, which remained stable at 4 °C for several days. It is necessary to note, however, that the refolded MucB protein was stable only in the refolding buffer and only at 4 °C or when kept on ice. A shift to room temperature or 37 °C resulted in a visible aggregation. While further optimizing the process of refolding of MucB protein on a gel filtration column, we found that the replacement of L-arginine hydrochloride with L-arginine and adjusting the pH with acetic acid instead of hydrochloric acid improves the refolding efficiency so that the LMW peak dominates over the HWM peak. The LMW fraction of MucB was used in all our subsequent studies. The yield of this method was similar to the method for purification of MucA’ protein: about 4.5 mg of refolded MucB in the LMW fractions per 500 ml of the induced culture originally containing about 100 mg of total cell proteins.

### Characterization of the purified MucA’ and MucB proteins by SPR

Because the refolded MucB protein aggregated upon exchange of its refolding buffer for another buffer, we could not efficiently analyze its properties by conventional biochemical methods. Thus, we chose an alternative and relatively new method, i.e. the Surface Plasmon Resonance (SPR). Using this method, we examined the interactions between MucB and MucA’ and between MucB and RecA. In addition, we tested whether MucB interacts with the β subunit of DNA polymerase III holoenzyme of *E. coli*, which is a ‘sliding clamp’ that encircles DNA and anchors the polymerases to the template DNA.

**Fig. 4:**
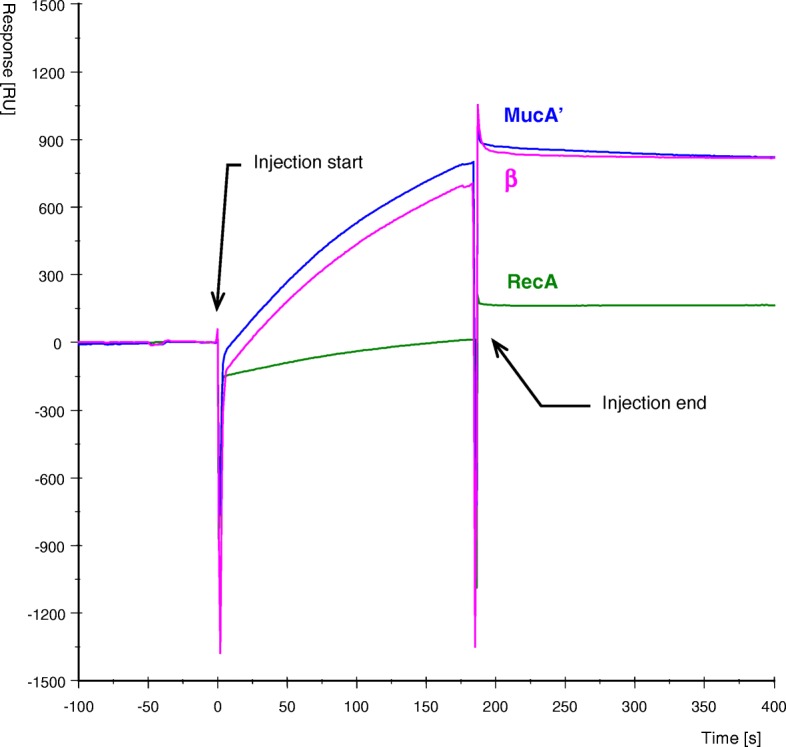
SPR analysis of interactions of MucB protein with MucA’, β and RecA proteins. The refolded MucB protein was injected simultaneously over the surface of four flow cells of the CM5 sensor chip immobilized with different proteins as described under “Experimental Procedures”. The increase in response signal [RU] during injection shows the kinetic of binding to the surface and the difference in signal before and after injection reflects the amount of MucB protein bound to the surface. The presented data are curves adjusted by subtracting the response curve in the control blank cell with immobilized BSA, to which MucB protein showed no binding, using the Pharmacia Biosensor kinetics software (BIAevaluation 2.1)

**Fig. 5:**
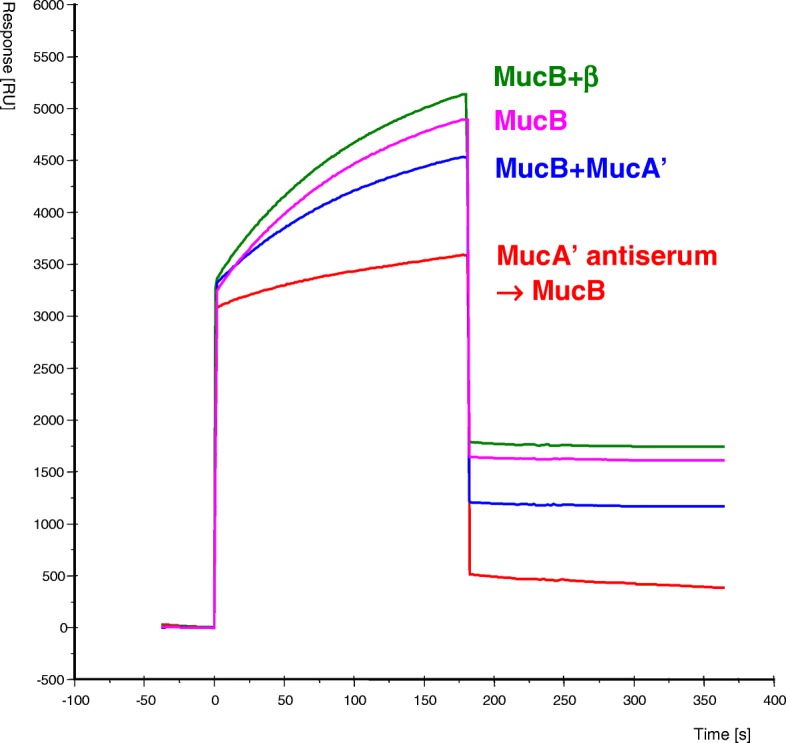
Specific inhibition of the MucB-MucA’ protein interaction. Similar amounts of MucA’ protein were immobilized on the surface of CM5 sensor chip in all four flow cells (Fc1, 7141 RU; Fc2, 6730 RU; Fc3, 6782 RU; Fc4, 6491 RU). The analysis was performed sequentially in separate flow cells in the order Fc4, Fc3, Fc2 and Fc1 as follows: Fc4, only MucB (0.12 μM) was injected; Fc3, MucB (0.12 μM) premixed with MucA’ (0.15 μM as a dimmer) were injected; Fc2, MucB (0.12 μM) premixed with β-subunit (0.3 μM) were injected; Fc1, MucB (0.12 μM) was injected over the surface pretreated with 10x diluted MucA’ antiserum resulting in binding of 7813 RU of MucA’ antibodies to the surface. The sensograms on the figure are labeled with the appropriate protein combinations injected over the corresponding flow cell surface

MucB protein was passed in the mobile phase over flow cells of the sensor chip containing BSA, RecA, MucA’ or the β-subunit, and the interactions between the analyte and the ligand, i.e., MucB versus RecA, MucA’ or the β-subunit, were determined by SPR*.* It turned out that the purified MucB protein quickly interacted with MucA’ and the β subunit, and slowly interacted with RecA (Fig. 4). MucB did not bind to BSA-coated or intact CM5 chip surface at all. To further check for the specificity of the interactions we did a control experiment (Fig. 5) which showed that the MucB interaction with MucA’ surface is prevented by MucA’ antibodies as well as inhibited by coinjection of MucA’ free in solution with MucB. Interestingly, coinjection of β-subunit free in solution with MucB did not decrease MucB binding to MucA’ surface suggesting that there are two distinct binding sites for MucA’ and β-subunit on a MucB protein molecule. We also examined the possible interaction between MucA’ and RecA by SPR. RecA and MucA’ were used at first as the analyte in a mobile phase and the ligand on a sensor chip, respectively, and then the positions of the two proteins were reversed. However, we could not observe any interactions between the two proteins regardless of the position of the proteins. We did not observe any interactions either when MucB was bound on the sensor chip, and RecA, MucA’ and the β-subunit were used as analytes.

Because the interaction between MucB and the β-subunit is new, and may play an important role in the mechanism of translesion DNA synthesis, we compared the affinity between MucB and the β subunit with that between MucB and MucA’ by calculating the kinetic parameters. For the purpose of calculating kinetic constants we conducted another experiments with lower ligand densities to avoid the potential error caused by re-binding of dissociating molecules to the chip due to the high densitiy of bound analyte. In these experiments (data not shown) we used 577 RU of immobilized β and 1036 RU of immobilized MucA’. The amounts of MucB protein bound to these surfaces during analysis was 349 RU for β and 355 RU for MucA’ and the calculated residual and lag plots for the kinetic analysis indicated good fit. Under these assay conditions, MucB protein showed higher affinity for the β-subunit than for the MucA’ protein (Table 1). This also corresponds to our observation that MucB protein could be only partially removed from the chip surface containing the β subunit by injection of 10 mM glycine pH 2.2, while such treatment completely regenerated the surface containing MucA’. For comparison, in another set of experiments, the calculated KD for the MucB and RecA interaction was 2.41 nM indicating weaker binding.

**Table 1:** Apparent kinetic constants for binding of MucB to MucA’ and β subunit of DNA polymerase

Mobile phase	Immobilized ligand	*k*_a_ [M^−1^ s^− 1^]	*k*_d_ [s^− 1^]	*K*_D_ [nM]
MucB	MucA’	6.39 × 10^4^	1.27 × 10^− 4^	1.99
MucB	β	3.87 × 10^4^	1.2 × 10^− 5^	0.31

In SPR, a ligand, i.e., MucA’ or β, is immobilized on a sensor chip, and an analyte, in this case MucB, is injected over the chip surface in a mobile phase. If the ligand and analyte interact, the resulting increase in mass on the chip surface is detected and plotted as an increase in response units (RU) over time, allowing calculation of the apparent association rate constant (*k*_a_) value. After the injection of protein in the mobile phase is complete, buffer is passed over the chip, and the dissociation of the proteins is observed as a loss in mass over time from which the apparent dissociation rate constant (*k*_d_) value can be calculated. From these data, the equilibrium dissociation constant (*K*_D_) is obtained (*K*_D_ = *k*_d_/*k*_a_). In the calculation, we used the first flow cell with immobilized BSA as a reference cell because MucB protein did not show any binding to it similar to the intact CM surface. To determine the kinetic constants with highest accuracy we calculated the values at several different densities of immobilized ligands and selected the values with the best fits according to the residual and lag plots

**Fig. 6:**
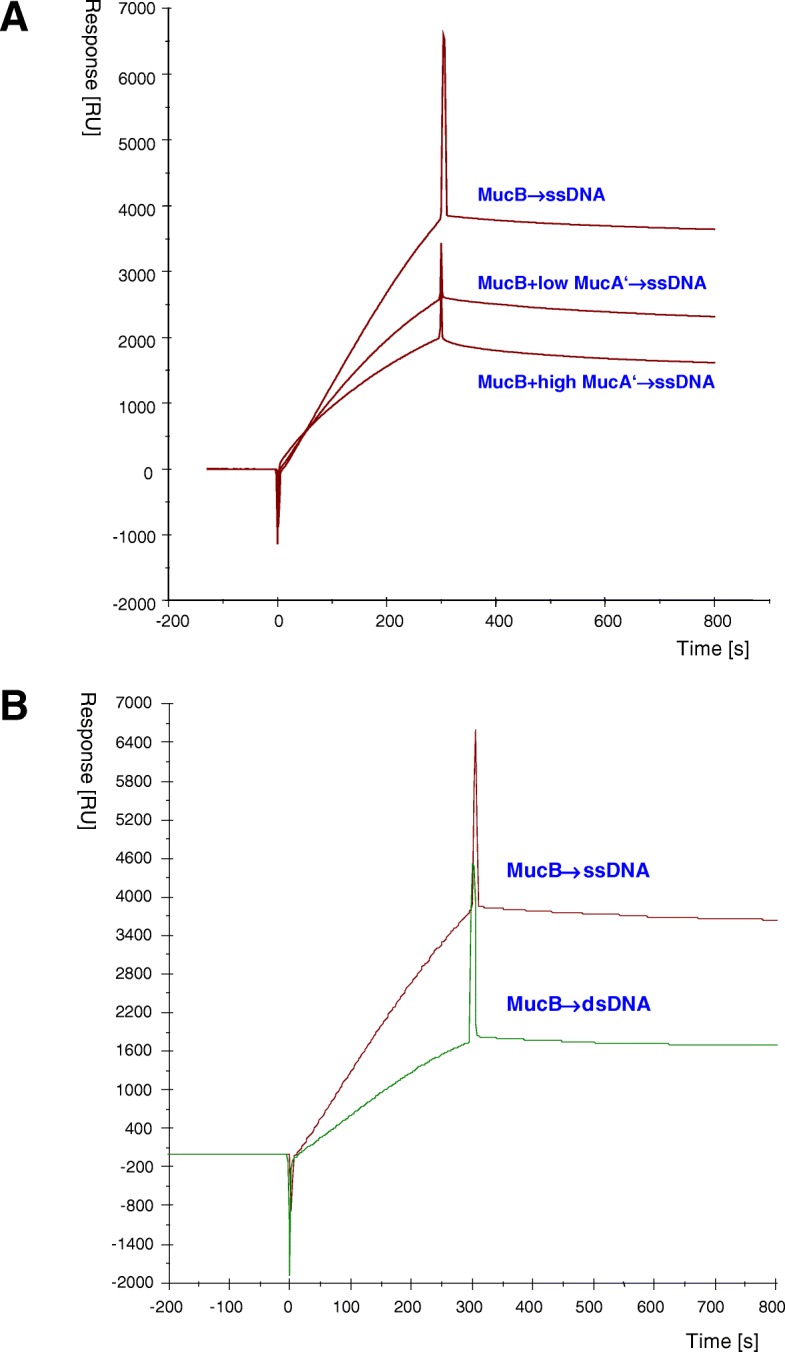
The effect of MucA’ on the binding of MucB to ssDNA. MucB (0.26 μM) alone or MucB (0.26 μM) premixed with MucA’ (1 μM or 10 μM, i.e. low or high, as indicated) proteins were injected over the flow cells of SA chip with immobilized 73-mer single- stranded oligonucleotide, double-stranded oligonucleotide and the control blank cell without DNA. When the binding to double stranded oligonucleotide was examined, the 73-mer single stranded oligonucleotide was first annealed with the complementary strand and then the annealed oligonucleotide was bound on the SA chip surface via streptavidin/biotin interaction. **a** Comparison of sensograms demonstrating the inhibitory effect of MucA’ on MucB binding to single stranded oligonucleotide. **b** Sensograms showing the kinetics of MucB binding to ssDNA and dsDNA. The presented data are curves adjusted by subtracting the response curve in the control blank cell streptavidin surface from those curves in the test flow cells using the Pharmacia Biosensor kinetics software (BIAevaluation 2.1) and therefore represent the DNA specific responses

Since both the purified UmuC protein, which was active in the in vitro replicative bypass of an abasic site, and the MucB protein, which was purified in denatured state and renatured by dialysis, have been reported to have ssDNA binding activity [27, 29, 30], we examined if the purified MucB protein also shows affinity for ssDNA. For this purpose, we employed the SPR technique as well to minimize artifacts related to instability of MucB protein. We bound a 73-mer oligonucleotide on the SA chip surface via biotin-streptavidin interaction and tested the binding of MucB alone or MucB premixed with MucA’ to this ssDNA. As shown in Fig. 6a, the purified MucB protein alone bound ssDNA. Interestingly, when MucB was premixed with MucA’, the binding efficiency to ssDNA decreased. Since in these experiments we noticed some background binding of MucB to the plain Streptavidin surface we also included the dsDNA oligonucleotide as a control. As seen on Fig. 6b, MucB protein had higher affinity for ssDNA than dsDNA. Unlike RecA, which requires Mg^++^ cation for the binding to ssDNA, MucB or MucB premixed with MucA’ did not require Mg^++^ cation for the binding to ssDNA and the binding was not influenced by ΑΤPγS (data not shown). We also tested the binding of MucA’ to ssDNA. However, the purified MucA’ protein did not bind to ssDNA (data not shown).

## Discussion

We have developed new methods for purification of MucA’ and MucB proteins from inclusion bodies in *E. coli*. The method for purification from insoluble inclusion bodies substantially simplifies whole procedure by using highly concentrated, relatively pure and proteolysis-resistant form of the protein as a starting material. The small size of MucA’ protein (13.6 kDa) and the previously reported success in the purification of MucA protein from inclusion bodies [45] encouraged us to proceed this way. The MucA’ protein indeed proved to be soluble and stable after refolding by stepwise dilution to the refolding buffer. We demonstrated that the purified MucA’ protein forms a molecular dimer in solution by gel-filtration method (Fig. 2). This is consistent with the reports that UmuD’, MucA and MucA’ form dimers in solution [13, 29]. In addition, the SPR analysis indicated that the purified MucA’ interacts with MucB (Fig. 4). These results suggest that the purified MucA’ and MucB proteins are biochemically active. We anticipate that the present purification method for MucA’ protein can be generally applicable to other UmuD’ homologues such as SamA’ or to the HisTag-derivatives of MucA’, which can be highly overproduced [38]. Since many proteins purified from inclusion bodies can yield crystal structures [44, 46] and the homologous UmuD’ protein has been successfully crystallized [24], we expect that the purified MucA’ could be crystallized for the determination of its structure. Because MucA’ constitues a part of the most active UmuD’C (Pol V) homologue, its further analysis should enlighten the structural bases important for the process of mutagenesis.

In contrast to the relative ease of purification of MucA’ protein, the purification of MucB has been a struggle similarly to its homologue, UmuC. So far it has been very difficult to isolate any close UmuC homologue in a concentrated soluble fully stable form. Even the whole DNA polymerase V (UmuC protein complexed with UmuD’) has been reported to aggregate at lower salt concentrations [30]. During and after the refolding of MucB protein, we had to include protein aggregation inhibitors, i.e.*,* L-arginine and Tween 20, in the buffer to prevent its aggregation. Arginine has been found to be a potent suppressor of protein aggregation [47]. Because of its formula similar to chaotopes like urea and guanidinium containing functional groups that can accept or donate hydrogen atoms in hydrogen bonding, it is likely that arginine labilizes misfolded or improperly associated molecules by competing favorably for weak nonspecific hydrogen bonds involved in the aggregate formation. Although arginine alone efficiently prevented aggregation of MucB, it was not effective on a longer time scale. It is reported that rhodanese, which serves as a model for refolding of insoluble proteins in vitro, can be efficiently refolded by “artificial chaperones” such as detergent micelles acting in similar manner as natural chaperones [48]. We therefore tested some low CMC nonionic detergents for their ability to prevent aggregation of MucB protein. Some of the tested detergents, such as Tween 20, indeed proved to inhibit aggregation of MucB protein. Finally, by a combination of L-arginine and the detergent Tween 20, we obtained the NAT refolding buffer, which not only prevented aggregation of MucB protein upon dilution from denaturant but also stabilized it in solution on a longer time scale. By conducting the refolding step on a gel filtration column, we could isolate a properly refolded low molecular weight form of MucB protein (Fig. 3).

The purified MucB protein allowed us to examine the possibilities that it interacts with other proteins involved in the mutagenesis process and with DNA. For this purpose, we used SPR method. The SPR analysis is widely used for real time and label free monitoring of biomolecular interactions, and is particularly useful for rapid and sensitive characterization of protein-protein and protein-DNA interactions [49–52]. From the protein-to-protein interaction studies, we observed that the purified MucB protein interacted with both MucA’ and RecA proteins (Fig. 4). This well corresponds to the interaction of MucA’ and MucB demonstrated by the two-hybrid system [29] and the behavior of its homologue UmuC, which is known to form complex with UmuD’ [14, 30]. The UmuC protein also interacted with RecA* immobilized on an affinity column [19] although the possibility that such an interaction could have been mediated through a third protein was not ruled out. In contrast to our finding, no interaction was found between MucB and RecA in the two-hybrid system [29]. Given the differences in the affinities between MucB and RecA versus MucA’ it is possible that the relatively low sensitivity and nature of the in vivo yeast two-hybrid system assay, compared to our in vitro BIAcore assay, do not simply allow efficient detection of the relatively weak interaction between MucB and RecA proteins.

Given the fact that UmuD’C proteins constitute a new DNA polymerase, i.e. DNA Pol V, and the close sequence as well as phenotypic similarities between the UmuD’C and MucA’B proteins, it is not surprising that the MucA’B proteins also constitute DNA replicative enzyme [53]. DNA polymerases as well as other enzymes involved in DNA metabolism are known to interact with the “sliding clamps” β subunit of DNA polymerase III in *E. coli* or PCNA in eukaryotes. Because the β,γ complex has been reported to be necessary for the DNA polymerization activity of Pol V [54] we examined whether the MucA’ and MucB proteins interact with the β subunit.

The β subunit of DNA polymerase III forms a homodimer with a toroidal structure [55], and tethers the polymerase to template DNA [56–58]. The presence of β subunit significantly increases the processivity of DNA polymerase III in *E. coli*. Interestingly, the SPR analysis indicated that the MucB protein interacts with β subunit of DNA polymerase (Fig. 4). The *K*_D_ value for the interaction was even smaller than that for the interaction between MucB and MucA’ (Table 1). Based on the results that MucB interacts with β subunit, we assume that MucA’B is at first targeted to ssDNA region close to a DNA lesion by binding to RecA; then it is transferred from RecA to β subunit when the DNA polymerase III dissociates from it at the damaged DNA region. We prefer the possibility that MucA’B is guided to the ssDNA region by binding to RecA rather than by directly binding to β subunit because the number of molecules per cell is about 80,000 for RecA when SOS is induced [59] and about 300 for β subunit [60]. However, we can not rule out the possibility that β subunit once bound to MucA’B might be recycled and slide along dsDNA until it encounters a ssDNA gap generated by the DNA polymerase III stalled at a replication blocking lesion.

PCNA, an eukaryotic counterpart of β subunit, is a trimer with a toroidal structure and increases the processivity of mammalian DNA polymerases δ and enhances the activity of DNA polymerase ε [61]. Because of the structural similarity between eukaryotic PCNA and the β subunit of *E. coli*, it is tempting to speculate that MucB might bind to PCNA as well. If so, the expression of MucA’B or MucAB could increase the ability for translesion DNA synthesis or the ability to promote mutagenesis in eukaryotic cells. Interestingly, it is reported that the introduction of mRNAs of MucA’B and UmuD’C enhances translesion DNA synthesis in *Xenopus* oocytes and oocyte nuclear extracts [62], and *mucAB* operon can stimulate mutagenesis in *Saccharomyces serevisiae* [63]*.* PCNA acts not only as a sliding clamp to increase the processivity but also as a modulator of cell cycle by interacting with other proteins such as cyclins, cyclin dependent kinases, FEN-1, Gadd45 and p21 [61]. In this respect, the report by Tosu and Tanooka [64] that the expression of *mucAB* induces cell transformation of Balb 3 T3 cells seems to be interesting.

Using the SPR technique, we also assayed the binding of MucB to ssDNA anchored on the chip surface as it has been reported that MucB bound ssDNA in a mobility shift assay [29]. Although we observed some binding to dsDNA as well, the results showed preferential binding of MucB to ssDNA (Fig. 6b). The observed binding of MucB to dsDNA might occur at regions of partially denatured structures such as breathing dsDNA termini or loops at A:T rich regions. The binding mechanism of MucB to ssDNA appears to be different from that of RecA to ssDNA because the binding of MucB did not require Mg^++^ cation and was not influenced by ATPγS. Generally, MucB binding to ssDNA was similar in affinity to that of RecA but weaker than that of SSB (Additional file 1: Figure S1), suggesting that MucB is unable to compete SSB from ssDNA. It is suggested that high-level of expression of UmuD’C results in binding to ssDNA even in undamaged cells and the binding impedes DNA replication, thereby inducing cell toxicity [30]. If similar things happened with MucA’B, MucA’ could be a modulator of MucB by depressing its binding to ssDNA as MucA’ coinjected over the ssDNA chip surface lowered MucB binding to ssDNA in a concentration dependent manner (Fig. 6a). It is also possible that the MucA’B complex binds ssDNA in a different manner than MucB alone e.g. by wrapping the DNA around the MucA’B complex versus forming a MucB-ssDNA filament resulting in different protein to DNA stoichiometry. The binding of MucB to ssDNA could be related to cell toxicity at high PolRI expression levels in vivo and might even impede primer extension by purified PolRI on a naked oligonucleotide in vitro. This could be also why we were unable to demonstrate an in vitro DNA polymerase activity with our MucB and MucA’ purified proteins even at the BIAcore analytical conditions (in HBS buffer at 25 °C) when MucB protein did not aggregate. For the polymerase activity to be seen, it may be necessary to first reconstitute and separate the MucA’-MucB complex from the MucB monomer or include other co-factors such as the γ-complex [53].

## Conclusions

We have purified the main components of the DNA polymerase RI, i.e. the MucA’ and MucB proteins, overexpressed in *E. coli* from inclusion bodies and developed new methods for their refolding to yield soluble non-aggregated proteins. The analysis of the refolded proteins by gel filtration and protein-protein and protein-DNA interaction analysis using the SPR technique indicated that the proteins behaved as expected for their native in vivo produced forms. Using the SPR method we have revealed that the MucB catalytical subunit strongly interacted with the processivity subunit of DNA polymerase III that implies its role as an accessory protein to DNA polymerase RI while it performs the translesion DNA synthesis. The MucA’ and MucB proteins prepared by the described method in large quantities may be suitable for various in vitro analytical purposes. DNA polymerase RI should be used preferably to other Y-family TLS DNA polymerases because of its outstanding potential to promote mutagenesis in vivo and large amount of data from the Ames tests.

## Additional file

Additional file 1: **Figure S1.** Comparison of MucB-ssDNA, RecA-ssDNA and Ssb-ssDNA interactions on the SM chip surface. (PDF 46 kb)

## Abbreviations

BSA: Bovine Serum Albumin
dsDNA: Double-Stranded DNA
HMW: High Molecular Weight aggregate
LMW: Low Molecular Weight form
MBP: Maltose Binding Protein
PCNA: Proliferating Cell Nuclear Antigen
RU: Response Units
SDS-PAGE: Sodium-Dodecyl-Sulfate Polyacrylamide Gel Electrophoresis
SPR: Surface Plasmon Resonance
SSB: Single Strand DNA Binding protein
ssDNA: Single-Stranded DNA
TLS: Translesion DNA Synthesis
