# Supplementary figures and images for: Purification and interactions of the MucA’ and MucB proteins constituting the DNA polymerase RI
Source: Genes Environ. 2019 May 2;41:10. doi: 10.1186/s41021-019-0125-8 (PMC6495647; doi:10.1186/s41021-019-0125-8)

Fig. S1

Comparison of MucB-ssDNA, RecA-ssDNA and Ssb-ssDNA interactions on the SM chip surface

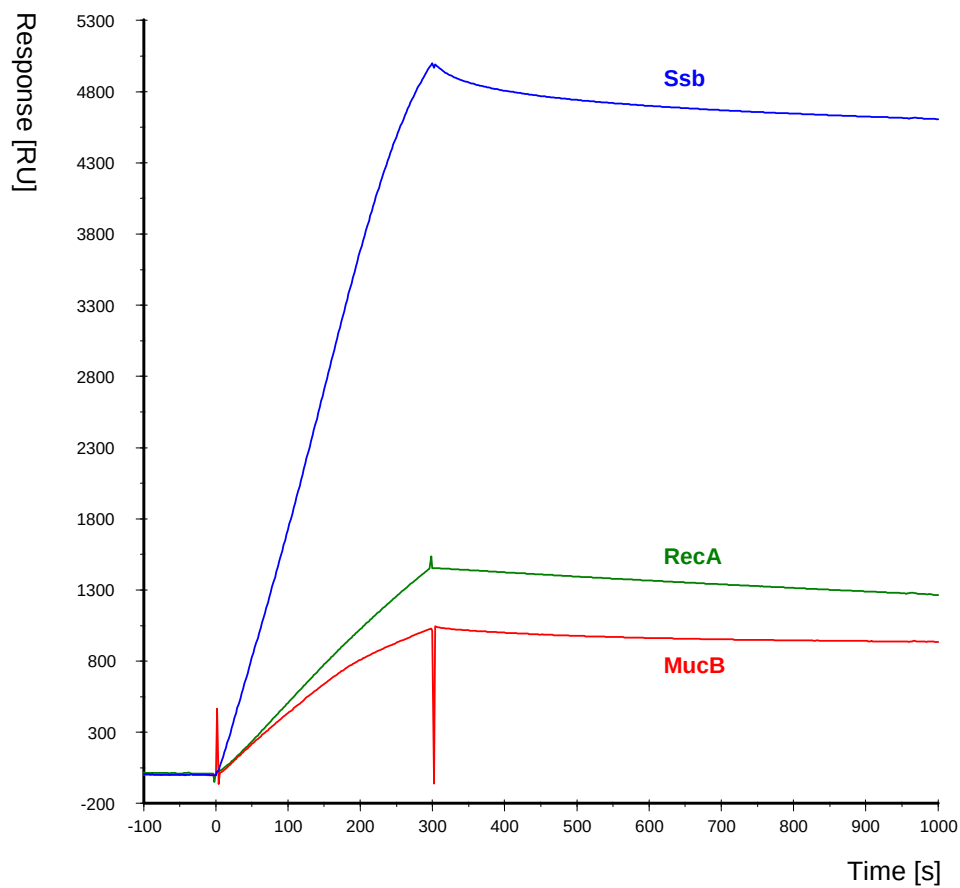

Supplement: Supplementary file 1 — Figure S1. Comparison of MucB-ssDNA, RecA-ssDNA and Ssb-ssDNA interactions on the SM chip surface. (PDF 46 kb) [file 41021_2019_125_MOESM1_ESM.pdf]
